# Metal-artifact reduced MR imaging for reverse shoulder arthroplasty: findings 1 year after surgery

**DOI:** 10.1007/s00256-025-05121-y

**Published:** 2026-01-21

**Authors:** Pia M. Jungmann, Martin Jaeger, Ferdinand C. Wagner, Balazs Bogner, Arsenij Molotkov, Thierno Diallo, Ralph Strecker, Reto Sutter, Fabian Bamberg, Matthias Jung

**Affiliations:** 1 Department of Diagnostic and Interventional Radiology, Medical Center - University of Freiburg, Faculty of Medicine, University of Freiburg, Hugstetter Strasse 55, 79106 Freiburg, Germany; 2 Department of Radiology, Kantonsspital Graubünden, Loëstrasse 170, 7000 Chur, Switzerland; 3 Department of Orthopedics and Trauma Surgery, Medical Center - University of Freiburg, Faculty of Medicine, University of Freiburg, Hugstetter Strasse 55, 79106 Freiburg, Germany; 4 EMEA Scientific Partnerships, Siemens Healthineers AG, Erlangen, Germany; 5 Department of Radiology, Faculty of Medicine, Balgrist University Hospital, University of Zurich, Forchstrasse 340, 8008 Zurich, Switzerland

**Keywords:** Shoulder, Arthroplasty, Postoperative, Magnetic resonance imaging (MRI)

## Abstract

**Objectives:**

To describe typical MRI findings 1 year after reverse total shoulder arthroplasty (rTSA) implantation.

**Materials and methods:**

Metal artifact reduction (MARS) MRI including CSSEMAC techniques was prospectively acquired in *N* = 25 consecutive patients (18/25 female; 61–80 years) 1 year after rTSA. MRI findings of bone and soft tissue and artifact reduction were assessed semi-quantitatively. Clinical Constant-Murley scores (CMS) were obtained, including its subscores pain, activities-of-daily-living (ADL), range-of-motion (ROM), and strength. Preoperative MRI was available in 13/25 subjects. Statistical analyses included descriptive statistics, Spearman correlations, nonparametric tests, and multivariable regression models.

**Results:**

MARS MRI of rTSA showed overall good image quality. Subacromial edema (88%), mild effusion (52%), and synovitis (44%) were frequent postoperative findings. Synovitis was associated with more pain (*B* = −2.044, 95% CI [−3.617, −0.470], *p* = 0.039) and lower strength (*B* = −4.497, 95% CI [−7.101, −1.893], *p* = 0.008). Minor bone marrow edema (BME) at the shaft was found in all subjects (100%). BME in Gruen zone 4 (88%) and 6 (80%) was most frequent. A higher number of Gruen zones with BME correlated significantly with lower ADL (*R* = −0.532, *p* = 0.024). There was a significant increase in fatty infiltration between pre- and postoperative images for the midacromial deltoid muscle (1 (IQR, 1–1) versus 2 (IQR, 1–2), *p* = 0.008; postoperatively present 100%). It was associated with an increase in deltoid length (chi-square 4.35, *p* = 0.037), but not with inferior clinical scores. However, fatty infiltration of the more anterior deltoid muscle was associated with lower ADL (*B* = −3.064, *p* = 0.008).

**Conclusion:**

Subacromial edema, minor shaft BME, and fatty infiltration of the midacromial deltoid muscle were typical, asymptomatic MRI findings 1 year after rTSA.

**Supplementary Information:**

The online version contains supplementary material available at 10.1007/s00256-025-05121-y.

## Introduction

The shoulder joint is the third most frequently replaced joint of the human body [1–3]. The implantation rate of reverse total shoulder arthroplasty (rTSA) has surpassed that of anatomical hemiarthroplasty [4]. Primary indications for rTSA include rotator cuff arthropathy, irreparable fractures in older patients, and failed anatomic arthroplasty [5]. Normal deltoid muscle function is required for rTSA since the deltoid muscle becomes the primary muscle driver for arm motion and joint stability [6, 7]. Despite overall satisfying postoperative outcomes, new complaints may occur during the further course [8–10]. If clinical and conventional radiography fail to identify a cause [11–13], metal artifact reduction (MARS) magnetic resonance imaging (MRI) can be applied [14–18]. Advanced metal artifact reducing techniques, including slice encoding for metal artifact reducing techniques (SEMAC) combined with advanced acquisition and reconstruction compressed sensing (CS) techniques, result in good image quality with acceptable scan times [14, 15, 17, 19–24]. However, achieving appropriate image quality at the shoulder joint is challenging. The shoulder joint is eccentrically located in the B0 field, is affected by breathing-associated motion artifacts and has a small soft tissue surrounding [17, 22, 25–27]. While empirical studies describe postoperative complications after shoulder arthroplasty [22, 27], no systematic postoperative MRI study has evaluated morphologic findings after rTSA [27, 28]. Therefore, the purpose of this study was to describe typical MRI findings 1 year after reverse total shoulder arthroplasty (rTSA) implantation.

## Methods

### Subjects

Individuals were prospectively and consecutively included between March 2021 and January 2023 on the occasion of the regular 12-month follow-up after rTSA implantation. Inclusion criteria were implantation of rTSA 12 months earlier and age ≥ 18 years. Symptomatic patients with self-presentation due to complaints were not included. Exclusion criteria were refusal of participation or MRI contraindications including cardiac pacemaker, cochlear implant, pregnancy, and claustrophobia. Informed consent was obtained from all individual participants included in the study.

### Treatment

All rTSA implantation procedures were performed by a single orthopedic surgeon using a standard deltopectoral approach (MJae, 20 years of experience). Arthroplasties analyzed included Medacta Reverse (*n* = 14; Medacta International SA, Castel San Pietro, Switzerland), Arthrex Univers ReversTM (*n* = 6; Arthrex GmbH, Munich, Germany), and Mathys Affinis Inverse (*n* = 5; Mathys AG, Bettlach, Switzerland). The supraspinatus tendon was cut. The infraspinatus tendon was reinserted. The postoperative rehabilitation protocol included limited shoulder abduction to 90° for 3 weeks with following careful mobilization. After week 6, full range of motion (ROM) was allowed.

### Image acquisition and analysis

Preoperative and 1-year follow-up conventional radiography included anteroposterior and lateral views of the shoulder. One year after rTSA implantation, MARS MRI of the shoulder was performed at a 1.5 T MR scanner (MAGNETOM Avanto Fit; Siemens Healthineers, Forchheim, Germany) using an 18-channel body coil. The MARS MRI protocol (Table 1) included a coronal and a transverse CSSEMAC short τ inversion recovery (STIR) sequence (vendor-specific research software package) with 15 spectral encoding steps, using 10 iterations for the compressed sensing reconstruction at a normalization factor of 0.001. Preoperative MRI of the shoulder was available in 13/25 subjects. Images were transferred to Picture Archiving Communication System workstations (Deep Unity, Dedalus HealthCare). Image assessment was performed by two radiologists in consensus with 16 (reader 1) and 10 (reader 2) years of experience in musculoskeletal imaging (PMJ; TD), respectively. Interclass correlation coefficients (ICC) for the shoulder osteoarthritis severity (SOAS) score were published earlier [29]. To determine intra- and interreader reliability for findings not included in the SOAS score, postoperative MR image sets were evaluated twice by reader 1 and reader 2. There was a minimum interval of 6 weeks between the reading sessions.

**Table 1:** MRI parameters

Parameter	Coronal CSSEMAC STIR	Transverse CSSEMAC STIR	Transverse VAT STIR	Transverse VAT T2w	Coronal VAT T1w	Sagittal VAT IMw
Repetition time (ms)	8350	8350	5570	6380	522	4390
Echo time (ms)	26	26	59	103	8.6	51
Inversion time	160	160	160	-	-	-
Echo train length	15	15	8	15	3	15
Averages	1	1	1	1	1	1
Number of slices	32	32	45	42	32	30
Thickness (mm)	4	4	4	4	3	4
Spacing (mm)	4	4	4.4	5	3.6	4.8
Matrix	256 × 205	256 × 205	320 × 256	384 × 269	512 × 461	384 × 269
Field of view (mm^2^)	240 × 240	200 × 200	220 × 220	199 × 199	240 × 240	229 × 229
Pixel size	0.94 × 0.94	0.78 × 0.78	0.69 × 0.69	0.52 × 0.52	0.47 × 0.47	0.60 × 0.60
Bandwidth (Hz/pixel)	500	500	300	500	515	500
MARS technique	CSSEMAC	CSSEMAC	VAT	VAT	VAT	VAT
Slice encoding steps	15	15	-	-	-	-
Flip angle (°)	140	140	150	170	150	170
Phase encoding direction	col	col	col	col	col	row
Acquisition time (min:s)	6:40	6:40	5:50	2:14	2:10	1:32

*MARS*, metal artifact reducing sequences; *CSSEMAC*, compressed sensing slice encoding for metal artifact correction; *STIR*, short tau inversion recovery; *VAT*, view-angle tilting; *T2w*, T2 weighted; *T1w*, T1 weighted; *IMw*, intermediate weighted

#### Conventional radiography

Deltoid length was measured between the acromion and the deltoid tuberosity of the humerus [30]. Lateral humeral offset (LHO) was measured from the center of rotation to the most lateral point of the greater tubercle [31]. Acromion-tubercle distance (ATD) was measured as the shortest distance between the acromion and the greater tubercle [32]. Heterotopic ossification (Brooker grade 1 to 3) was noted [33].

#### MARS MRI evaluation

Metal artifacts on MARS MRI were evaluated using a 5-point Likert scale (1 = worst, 5 = best) as follows: (i) overall metal artifact reduction; (ii) motion artifacts; (iii) overall image quality; (iv) artifact reduction, ripple artifacts, soft tissue image quality, and overall image quality for CSSEMAC STIR and view-angle tilting (VAT) STIR images.

Following the SOAS score [29], MRI evaluation on a semiquantitative scale included synovitis (0 = none to 2 = severe), joint effusion (0 = none, 3 = severe), subacromial edema/fluid (0 = none, 3 = severe), and fatty infiltration (Goutallier 0 to 4 [34])/atrophy (Thomazeau 0 to 3 [35]) of rotator cuff muscles. In addition, edema of the individual rotator cuff muscles (0 = none, 3 = severe) was assessed. Periarticular fluid and lamellar synovitis were noted. The deltoid muscle was evaluated regarding fatty infiltration (Goutallier score [34]), atrophy (Thomazeau score [35]), and edema (0 = none, 3 = severe) in its clavicular, acromial (anterior, middle, and posterior), and spinal part. Additionally, specific parameters to evaluate findings after rTSA were assessed, including bone marrow edema (BME; 0 = none, 3 = severe) at the acromion, scapula, and coracoid process. For adapted Gruen and Molé zones (Supplemental Material S1), BME, periprosthetic resorption, periosteal edema, and periosteal reaction were assessed [12, 33, 36–38].

### Clinical assessment

The Constant-Murley score (CMS) was obtained 1 year after rTSA implantation [39]. The 100 point system (100 = best, 0 = worst) consists of four categories: pain (15 points), activities of daily living (ADL; 20 points), strength (25 points), and ROM (40 points) [39]. More points indicate better shoulder function: < 30 unsatisfactory, 30–39 fair, 40–59 good, 60–69 very good, and ≥ 70 excellent [39]. Isometric strength measurements were performed using a digital dynamometer (IsoForceControl® EVO2 Mobile Dynamometer, Medical Device Solutions). Maximal isometric strength (in kilograms) was assessed bilaterally via Jobe and Starter tests (abduction) and external rotation with 33 measurements per second for a 5-s period [40].

### Statistical analysis

Statistical analysis was performed by one radiologist (PMJ) using SPSS v26 (IBM, Armonk, NY, USA). A priori power analysis was conducted using GPower 3.1 (*α* = 0.05). Sample size estimation based on the SOAS score difference between patients with and without osteoarthritis indicated that a total sample size of 12 participants would provide a statistical power of 0.958. Primary outcome parameters were the CMS subscores. A linear regression model was applied to exclude an influence of age, gender, and body mass index on the primary outcome parameters. Due to the small sample size, statistical analyses included descriptive statistics, nonparametric Wilcoxon signed-rank tests (for paired analyses), Mann-Whitney *U* tests (for two independent groups), and Spearman’s rho correlations. Multivariable stepwise linear regression models were used to identify postoperative MRI findings with significant influence on CMS subscores. Alpha was set at 0.2. As level of significance 0.05 was assumed for all tests. To control for multiple testing (four CMS subscores),* p*-values were corrected using the Holm-Bonferroni method. Frequency values are presented as median (interquartile range (IQR)). For intra- and interreader reliability, ICC were calculated [41, 42].

## Results

### Subjects and clinical findings

A flowchart on patient selection is provided in the Supplemental Material S2. Subjects’ characteristics and clinical scores are provided in Table 2. The right shoulder was affected in 48% of cases (12/25). Postoperative follow-up times to clinical and MRI assessment were 12 (IQR, 11–14) months (12 (7.5–14) months to conventional radiograph). Preoperative radiographs were performed 1 (0–2) months before surgery. A subset of 13/25 individuals had preoperative MRI (9 (4.5–9) months before surgery). One year after surgery, 15/25 individuals had an excellent CMS score ≥ 70. Maximum isometric strength was significantly lower ipsilateral versus contralateral for external rotation (4.1 (IQR, 3.2–6.4) kg versus 5.7 (4.5–6.7) kg, *p* = 0.018) and Jobe test (5.0 (4.2–6.8) kg versus 6.5 (5.1–8.3) kg, *p* = 0.045).

**Table 2:** Clinical parameters

	**Range**	**Median (IQR)**			*p*-value*
		**Total cohort (*n* = 25)**	**Male (*n* = 7)**	**Female (*n* = 18)**	
Age (years)	61 to 80	74.5 (69.5, 79.3)	70.9 (69.3, 76.8)	76.0 (69.6, 79.5)	0.304
BMI (kg/m^2^)	18.6 to 40.8	28.7 (24.1, 29.7)	28.7 (25.0, 29.4)	27.7 (23.0, 30.6)	0.904
	**Possible range of points (worst to best)**	**Median (IQR)**			*p*-value*
		**Total cohort (*n* = 25)**	**Male (*n* = 7)**	**Female (*n* = 18)**	
Total CMS	0 to 100	77.0 (64.0, 81.5)	68.0 (53.0, 88.0)	77.5 (66.0, 80.8)	0.785
CMS subscores:					
Pain	0 to 15	13 (10, 15)	15 (11, 15)	13 (10, 15)	0.462
ADL	0 to 20	18 (15, 20)	18 (17, 19)	18 (14.3, 20)	0.951
Strength	0 to 25	10 (9, 15)	9 (7, 16)	10.5 (9, 15)	0.562
ROM	0 to 40	30 (24, 34)	26 (18, 34)	31 (26, 34)	0.501

*IQR*, interquartile range; *BMI*, body mass index; *CMS*, Constant-Murley score; *ADL*, activities of daily living; *ROM*, range of motion *p-values comparing male and female values based on Mann-Whitney *U* tests

### Conventional radiography

The causes for implantation of the rTSA were posttraumatic pseudarthrosis of the proximal humerus (7/25), osteoarthritis including cuff arthropathy (17/25), and failed anatomical ellipsis prosthesis (1/25). Postoperatively, most individuals (16/25) had heterotopic ossifications, most frequently in the axillary recess (*n* = 11).

### Metal artifact reduction

**Fig. 1:**
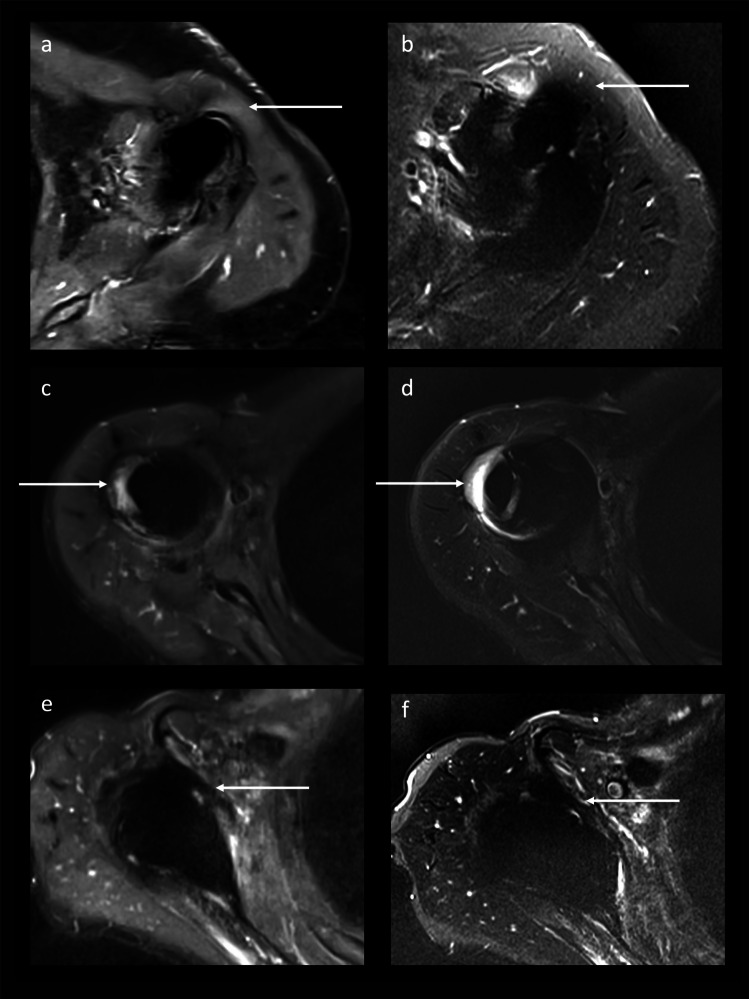
Soft tissue image quality on transverse CSSEMAC STIR images versus transverse VAT STIR images. Distant to the implant, the potential muscle edema on CSSEMAC (**a** 74 years, female) was identified as artifact when comparing to VAT STIR images (**b** same subject as **a**). While definitely being identified as greater tubercle bone marrow edema on CSSEMAC (**c** 75 years, female), the area was obscured by artifacts on VAT STIR images (**d** same subject as **c**). The subscapularis tendon was identified more easily as intact on CSSEMAC images (**e** 80 years, male) than on VAT STIR images (**f** same subject as **e**). CS, compressed sensing; STIR, short τ inversion recovery; SEMAC, slice encoding for metal artifact reducing techniques; VAT, view-angle tilting

**Fig. 2:**
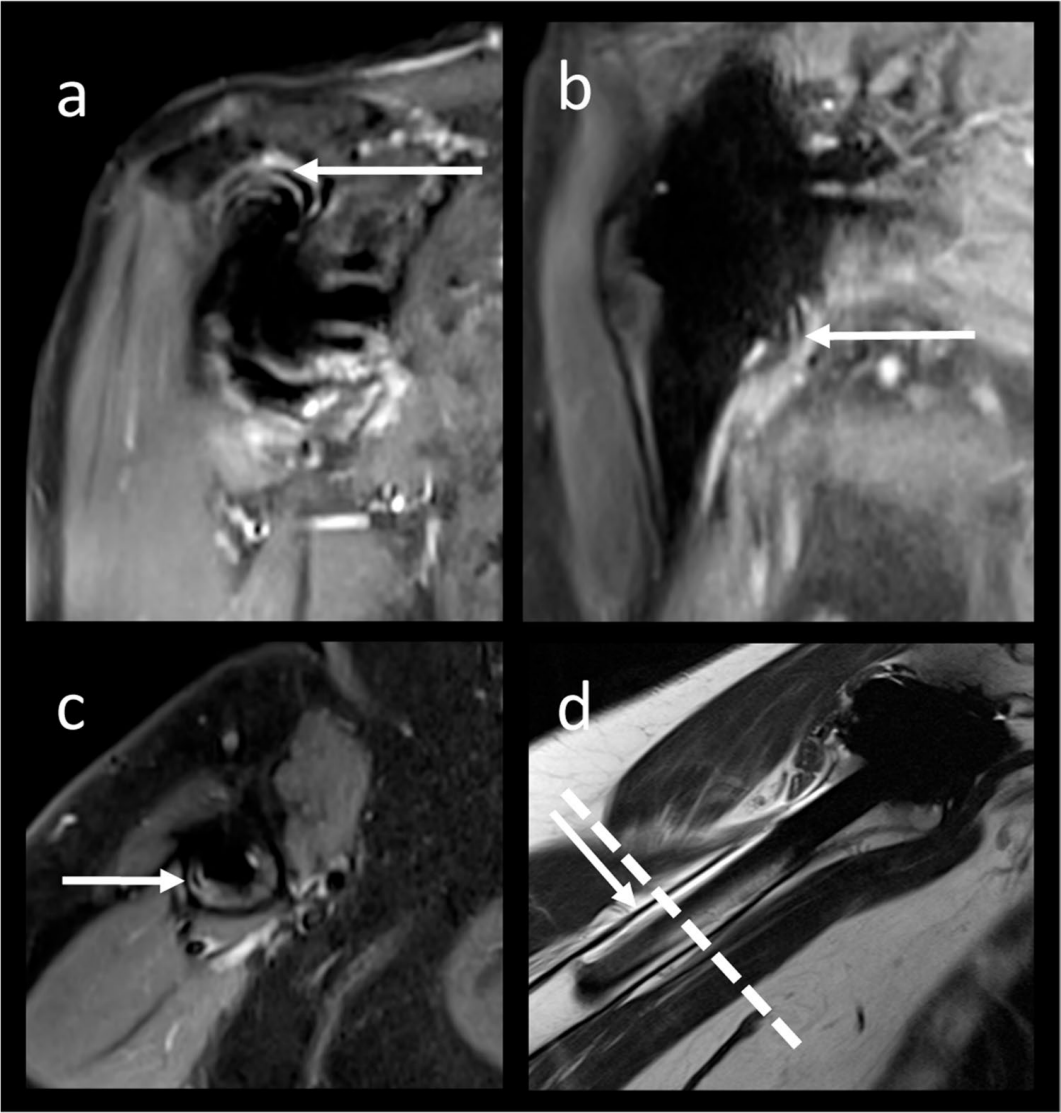
Ripple artifacts on CSSEMAC STIR images had a varying extent. Ripple artifacts were mainly found in the subacromial space (**a** 80 years, female) and at the axillary recess (**b** 77 years, female), not to be confused with lamellar synovitis. Ripple artifacts on transverse CSSEMAC STIR images (**c** 74 years, female) potentially imitated loosening and needed correlation with other sequences (sagittal IMw, **d** same subject as **c**). IMw, intermediate weighted; STIR, short τ inversion recovery; SEMAC, slice encoding for metal artifact reducing techniques

MARS MRI of rTSA demonstrated good artifact reduction (3 (IQR, 2–4); range 2–5) and good image quality (4 (IQR, 3–4); range 2–4; Supplemental Material S3a). Five MRI datasets showed motion artifacts. The image quality and metal artifact reduction were significantly better for CSSEMAC than for VAT (*p* = 0.007 and *p* < 0.001). Following, evaluation of periprosthetic tissue was easier on CSSEMAC, including the metal-bone interphase and the adjacent rotator cuff tendons (Fig. 1c–f). Although soft tissue image quality was not significantly different between the sequences (*p* = 0.346), findings distant from the implant could be better assessed on standard STIR VAT sequences than on STIR CSSEMAC in some cases (Fig. 1a, b). Ripple artifacts were present in CSSEMAC STIR images, mostly subacromial, at the axillary recess and/or at the shaft with different extent (range 1–5; Fig. 2), sometimes imitating lamellar synovitis (Fig. 2b). To exclude osteolysis, CSSEMAC images had to be correlated with other sequences (Fig. 2c, d).

### MRI findings

The reliability measures reached good (ICC > 0.75) to excellent (ICC > 0.9) agreement (Supplemental Material S3b). Postoperative MRI findings with frequencies > 75%, > 50%, and > 25% in the assessed cohort are indicated in Table 3 and Supplemental Material S4 in strong, medium, and low color intensity.

**Table 3:**
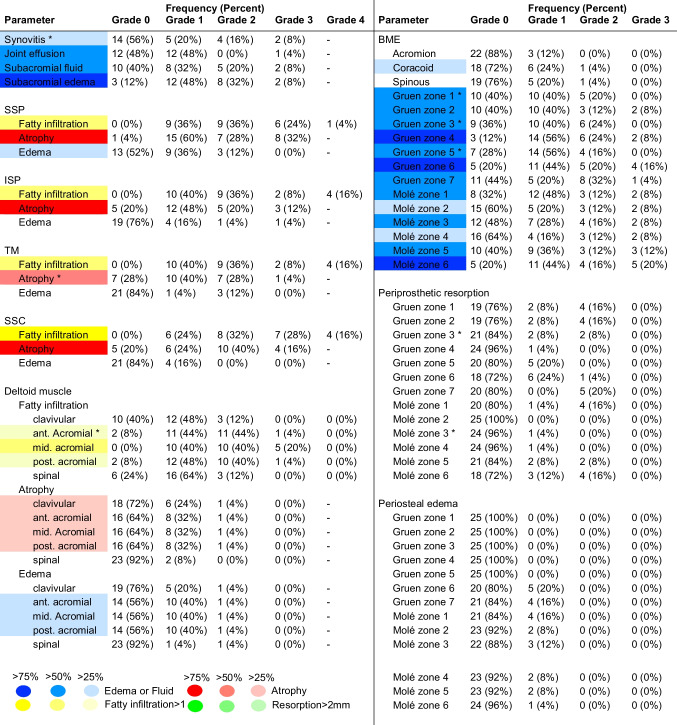
Frequency of postoperative MRI findings

*SSP*, supraspinatus muscle; *ISP*, infraspinatus muscle; *TM*, teres minor muscle; *SSC*, subscapularis muscle;* BME*, bone marrow edema. Asterisks indicate significant associations with clinical outcome scores

### Bone

All individuals showed at least minor BME in some region along the humeral stem. 18/25 (72%) had BME in 4 or more Gruen zones. Most frequently, BME was found in Gruen zones 4 (22/25; 88%) and 6 (20/25; 80%). Most individuals (23/25, 92%) showed at least minor BME in one region around the glenosphere; 12/25 (48%) had BME in 4 or more Molé zones. It was most frequently found in Molé zone 6 (19/25; 76%) followed by zone 1 (16/25; 64%). BME at the middle part of the acromion was found in 3/25 cases (12%); there was no acromial fracture. The three individuals with BME at the acromion had significantly higher strength scores (17 (IQR, 14–20) versus 10 (IQR, 9–14), *p* = 0.043). BME was present in the scapular spine and in the coracoid process in about one quarter of individuals (24% and 28%, respectively).

Periprosthetic resorption in at least one zone was found at the shaft in 8/25 (32%) individuals and at the glenoid in 7/25 (28%) individuals. Resorption > 2 mm in more than 2 zones at the shaft was found in 2/25 (8%) individuals (Glenoid 1/25; 4%). Resorption > 2 mm was found most frequently in Gruen zone 7 (5/25; 20%), followed by zones 1 and 2 (4/25; 16%) and Molé zones 1 and 6 (each 4/25; 16%). Periosteal edema was observed in 6/25 (24%) individuals in Gruen zones 6 and/or 7, respectively, and in 4/25 (16%) individuals around the glenoid. No subject showed periosteal reaction.

A higher number of Gruen zones with BME correlated significantly with lower ADL scores (*R* = −0.532, *p* = 0.006, adjusted *p* = 0.024; lower, upper 95% confidence interval (CI) [−0.756, −0.151]). A higher number of Gruen zones with resorption > 2 mm correlated significantly with lower ROM (*R* = −0.410, *p* = 0.042, adjusted *p* = 0.168; 95% CI [−0.704, −0.016]).

### Effusion and synovitis

Effusion (present in 13/25 (52%), 12/13 small) and synovitis (present in 11/25 (44%), 5/11 minor) were frequent postoperative findings. No subject had lamellar synovitis or periarticular fluid collections.

### Deltoid muscle

One year after rTSA implantation, atrophy of the deltoid was found most frequently in the acromial deltoid muscle (9/25; 36%), followed by the clavicular part (7/25; 28%). Similarly, most deltoid muscle edema was found in the acromial part (11/25; 44%), followed by the clavicular part (6/25; 24%). Rarely (*n* = 2, 1/2 small), edema was found in the spinal deltoid part. Fatty infiltration of the deltoid was found in nearly all individuals in all parts of the acromial deltoid muscle (anterior 23/25, 92%; middle 25/25, 100%; posterior 23/25, 92%), of which about 40 to 48% was Goutallier Grade 1 (clavicular deltoid fatty infiltration: 15/25, 60%; spinal deltoid fatty infiltration: 19/25, 76%). There was a significant increase in deltoid fatty infiltration between preoperative and postoperative MRI (*n* = 13) for the clavicular deltoid muscle (0 (IQR 0–0) versus 1 (0–1), *p* = 0.035) and for the anterior (1 (0–1) versus 1 (1–2), *p* = 0.025), middle (1 (1–1) versus 2 (1–2), *p* = 0.008), and posterior (1 (0–1) versus 1 (1–2), *p* = 0.020) acromial deltoid muscle, but not for the spinal deltoid muscle (0 (0–1) versus 1 (0.5–1), *p* = 0.096). Individuals with an increase in fatty infiltration in the midacromial deltoid (61.5%) had a higher increase in deltoid length from baseline to follow-up (chi-square 4.35, *p* = 0.037).

### Subacromial edema and fluid

Subacromial edema and/or fluid was a postoperative finding present in nearly all individuals (24/25; 96%). Severity varied between mild and severe. Subacromial fluid was more pronounced in individuals with a higher ATD difference between baseline and follow-up (*R* = 0.532, 95% CI [0.119, 0.808], *p* = 0.008) and those with a higher postoperative ATD (*R* = 0.442, 95% CI [0.070, 0.733], *p* = 0.031).

### Rotator cuff

One year after surgery, fatty infiltration and atrophy varied between grades 1 and 4. Edema was observed most frequently in the supraspinatus muscle (SSP; 12/25). Fatty infiltration > grade 2 was found in 28% (SSP), 24% (infraspinatus muscle (ISP)/teres minor muscle (TM)), and 44% (subscapularis muscle (SSC)). In the *n* = 13 subjects with preoperative MRI, the increase of fatty infiltration between preoperative MRI and 1 year postoperative MRI was significant for the SSP (preoperative 1 (IQR, 1–2) versus postoperative 2 (1–3), *p* = 0.014), ISP (1 (0.5–1.5) versus 2 (1–2.5), *p* = 0.010), and SSC (1 (1–1.5) versus 2 (1.5–3), *p* = 0.003). Atrophy increased for the SSP and ISP (*p* < 0.05).

### Multivariable linear regression models

**Fig. 3:**
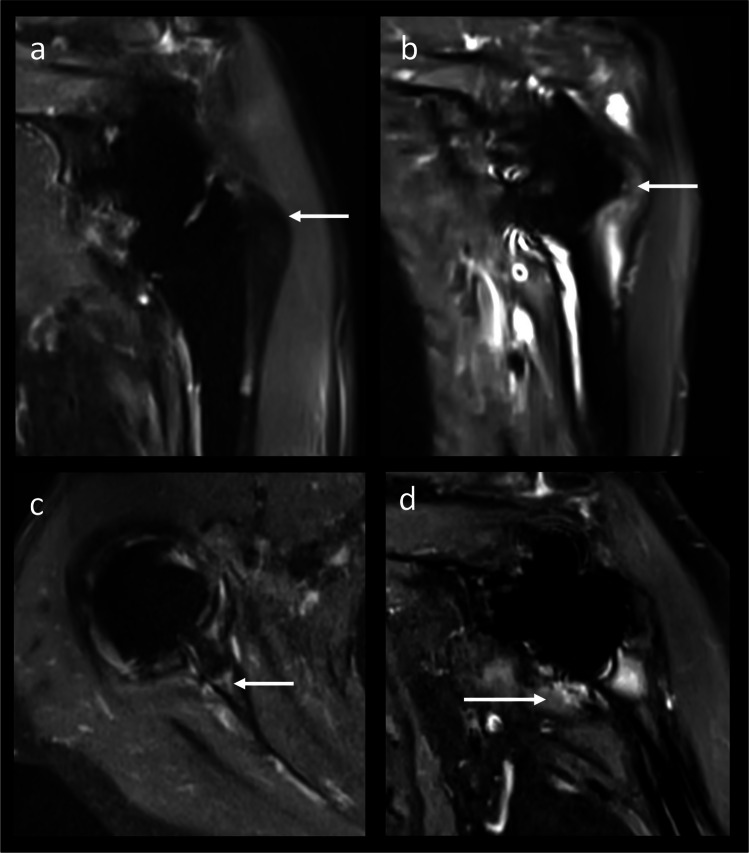
MRI findings associated with inferior clinical outcome measures 1 year after rTSA. In contrast to no BME at the greater tubercle (Gruen zone 1; **a** 70 years, male), subjects with severe BME at the greater tubercle (**b** 75 years, female) and those with BME in the medial glenoid (Molé zone 3; **c** 80 years, female) had inferior clinical outcomes. Synovitis (**d** 69 years, female) was a frequent finding, but was associated with inferior clinical outcomes. BME, bone marrow edema; rTSA, reverse total shoulder arthroplasty

**Fig. 4:**
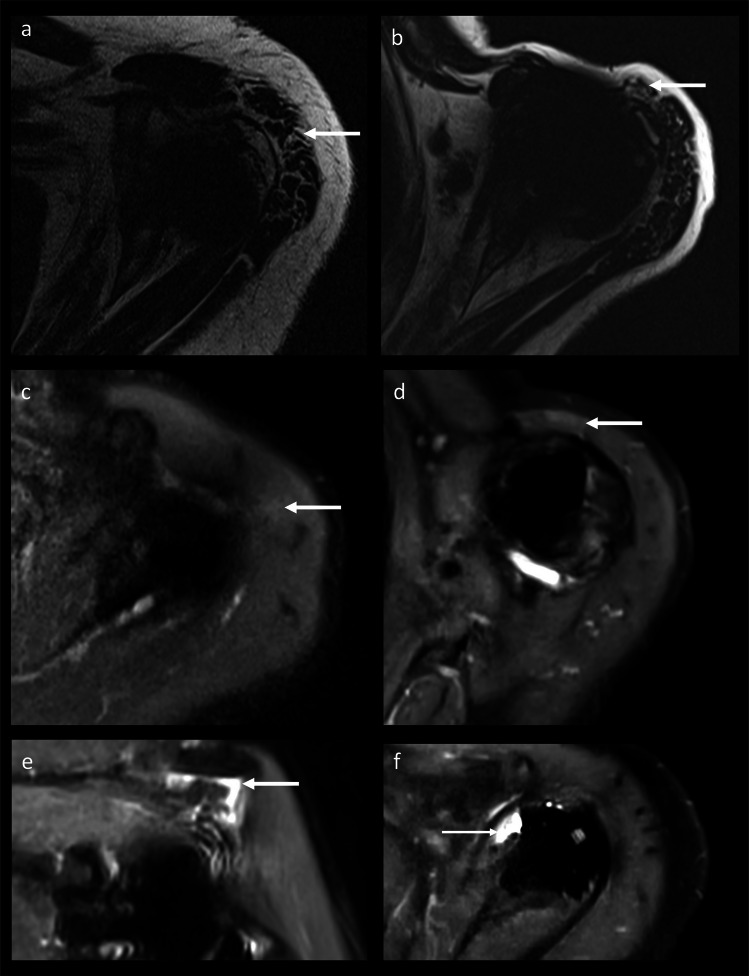
Deltoid muscle and subacromial changes. Typical fatty infiltration accentuated in the middle acromial deltoid muscle portion (**a** 79 years, female) was progressive from pre- to postoperatively, and the change was associated with deltoid length increase but not with inferior clinical outcomes. More anteriorly located fatty infiltration (**b** 77 years, female) was associated with inferior clinical outcomes. While edema of the middle acromial part was a typical postoperative finding without clinical relevance present in > 40% of cases (**c** 70 years, male), edema and atrophy of the clavicular deltoid part (**d** 77 years, female) were more rare and may need to be interpreted with caution. Subacromial edema and fluid (**e** 70 years, male) were frequent postoperative findings, associated with a higher acromion-tubercle distance and were without clinical significance. Similarly, effusion (**f** 79 years, female) had no clinical significance. CMS, Constant-Murley score

**Fig. 5:**
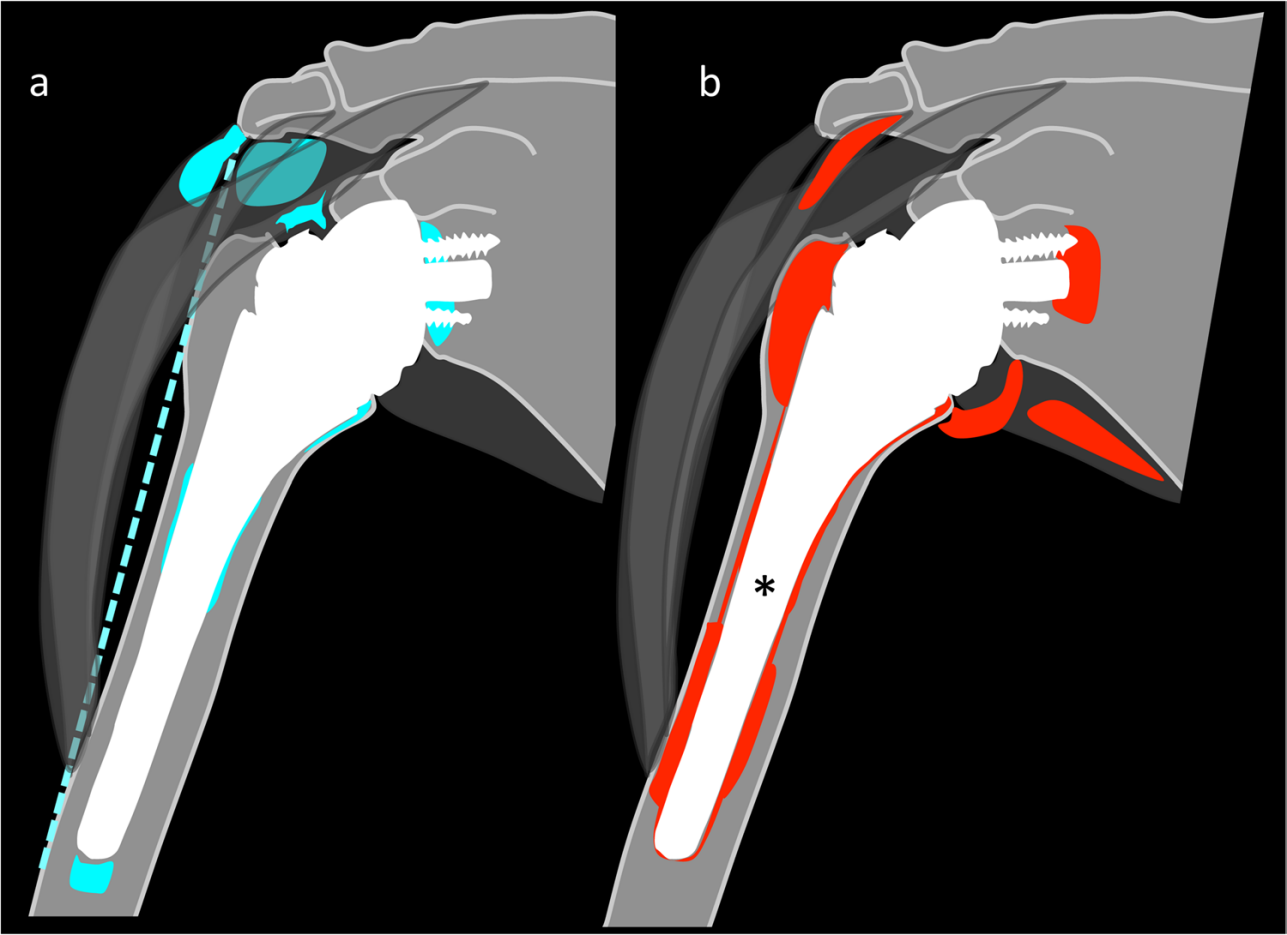
Schematic overview of typical asymptomatic findings (**a**) versus findings with potential clinical relevance (**b**) on MARS MRI 1 year after rTSA. **a** Findings with frequencies > 50% but without clinical associations were: subacromial edema and fluid, joint effusion, minor BME in Gruen zones 2, 4, 6, and 7 as well as at the Glenoid, and fatty infiltration of the midacromial deltoid muscle. Deltoid length was positively associated with strength. **b** Findings with worse clinical scores were as follows: BME in Gruen zones 1, 3, and 5, periprosthetic resorption in Gruen zone 3 and Molé zone 3, fatty infiltration of the anterior acromial deltoid muscle, teres minor atrophy, and synovitis. Further, the asterisk indicates that the number of zones with BME and the number of zones with periprosthetic resorption at the shaft correlated significantly with clinical outcome scores. BME, bone marrow edema; rTSA, reverse total shoulder arthroplasty

All variables were analyzed in multivariable linear regression models with subsequent Holm-Bonferroni correction. Postoperative MRI findings with negative significant influence on pain were BME in Gruen zone 1 (*B* = −1.870, 95% CI [−2.853, −0.886], *p* = 0.001, adjusted *p* = 0.004, Fig. 3) and synovitis (*B* = −2.044, 95% CI [−3.617, −0.470], *p* = 0.013, adjusted *p* = 0.039). Findings with negative significant influence on ADL were fatty infiltration of the anterior acromial deltoid muscle (*B* = −3.064, 95% CI [−4.818, −1.309], *p* = 0.002, adjusted *p* = 0.008) and BME in Gruen zone 3 (*B* = −2.445, 95% CI [−4.160, −0.729], *p* = 0.007, adjusted *p* = 0.028). Findings with significant influence on strength were delta length (*B* = 1.110, 95% CI [0.474, 1.746], *p* = 0.002, adjusted *p* = 0.008), BME in Gruen zone 1 (*R* = −1.831, 95% CI [−3.496, −0.166], *p* = 0.033, adjusted *p* = 0.099), and synovitis (*B* = −4.497, 95% CI [−7.101, −1.893], *p* = 0.002, adjusted *p* = 0.008). Findings with significant influence on ROM were fatty infiltration of the anterior acromial deltoid muscle (*B* = −2.991, 95% CI [−5.826, −0.156], *p* = 0.040, adjusted *p* = 1.20), atrophy of the TM (*B* = −3.784, 95% CI [−5.919, −1.649], *p* = 0.001, adjusted *p* = 0.004), BME in Gruen zone 5 (*B* = −3.353, 95% CI [−5.805, −0.900], *p* = 0.010, adjusted *p* = 0.040), and periprosthetic resorption in Gruen zone 3 (*B* = −9.047, 95% CI [−12.034, −6.060], *p* < 0.001) and Molé zone 3 (*B* = −3.777, 95% CI [−6.512, −1.041], *p* = 0.009, adjusted *p* = 0.036). The typical fatty infiltration of the midacromial deltoid muscle after rTSA (Fig. 4) was not associated with inferior clinical outcomes (*p* > 0.05). Findings with significant influence on CMS subscores are marked with asterisks in Table 3 and are visualized in Fig. 5 and Supplemental Material S4.

## Discussion

Optimized MARS MRI is essential for postoperative evaluation after rTSA. This study shows that several MRI findings can be considered physiological: these include minor BME, subacromial fluid or edema, small effusion, and fatty infiltration of the middle acromial deltoid muscle. However, fatty infiltration of the more anterior deltoid muscle was associated with inferior clinical outcomes. Other associations with inferior outcomes included synovitis, BME in Gruen zones 1, 3, and 5, and a higher number of periprosthetic BME or resorption zones at the shaft.

Shoulder arthroplasty is the third most commonly performed joint replacement [1]. Clinical outcomes after rTSA are generally good, as confirmed by median CMS of 77 in this cohort [8–10]. Nevertheless, complications remain relevant concerns [10, 43]. When symptoms are not explained by clinical findings and radiographs, MARS MRI is recommended [17, 23, 44, 45]. MARS MRI is well established for total hip arthroplasties (THA) [13, 15, 22, 23]. However, for shoulder arthroplasties, it is more challenging, due to the eccentric position of the shoulder, motion artifacts, and less periarticular soft tissue [26, 27]. In contrast to initial SEMAC sequences with very long acquisition times of 8–12 min, the introduction of compressed sensing has reduced scan times to 4–5 min, enabling clinical application [14, 22–24]. In this study, CSSEMAC STIR provided superior tendon visualization compared to VAT STIR, despite some loss of soft tissue contrast, confirming previous observations [23, 46]. Ripple artifacts were observed on CSSEMAC sequences in the subacromial space, axillary recess, or around the shaft. These artifacts had to be differentiated from loosening or periprosthetic fluid collections by comparison with VAT sequences and conventional radiographs. Ripple artifacts are common in (CS)SEMAC due to limited spectral fidelity of applied radio-frequency pulses [23, 47, 48].

Subacromial edema and fluid were present in most cases in the present cohort. While these findings indicate bursitis in native shoulders [29], after rTSA, the increased acromiohumeral distance likely leads to physiologic fluid accumulation or granulation tissue within the bursa [49].

In the present study cohort, minor humeral shaft BME and glenoid BME were found in most individuals after rTSA, similar to previously described findings after THA [14, 15]. In THA, the extent of BME reduces over time and is more prevalent in symptomatic individuals, paralleling the observations made for the shoulder in this study [14, 15]. BME was most frequent at the distal tip of the shaft (Gruen zone 4), which may reflect mechanical stress transfer. Interestingly, worse greater tubercle (Gruen zone 1) healing after rTSA was previously linked to inferior CMS and external rotation, which is in line with our finding that BME in Gruen zone 1 was associated with more pain and inferior strength [50]. Also in the osteoarthritis literature, mainly BME and synovitis are associated with clinical outcome measurements, particularly pain [51–54]. After cartilage repair, BME also predicts worse pain and outcomes [53, 54]. Bone resorption has previously been described typically for zones 1, 2, and 7 after rTSA, which could also be observed in this study [55].

After rTSA, the deltoid becomes the primary abductor of the shoulder joint [56–59]. The center of rotation is being medialized and distalized, which improves the deltoid lever arm, strength, and CMS [6, 7, 60]. While postoperative degeneration of the lateral deltoid muscle has been described, its impact on clinical outcomes remains controversial [5, 57, 61, 62]. In the present study, fatty infiltration of the midacromial deltoid muscle increased from pre- to postoperatively in the subset of participants with preoperative MRI. Individuals with an increase of this fatty infiltration had a higher increase of deltoid length from pre- to postoperatively. On the 1-year follow-up MRI, this fatty infiltration was present in 100% of individuals. Following, the pattern likely reflects physiological adaptation (fiber elongation induced fatty infiltration) rather than degeneration [63–65]. Similar changes occur after gluteal tendon repair without impairing function [66]. In contrast to the midacromial deltoid part, fatty infiltration of the more anterior acromial deltoid was negatively associated with clinical outcomes (ADL and ROM) in our study, which is consistent with Wiater et al. and other studies [5, 19, 67]. Deltoid edema was mostly located in the midacromial region, likely also reflecting mechanical adaptation. On the contrary, edema or atrophy in the clavicular delta part needs to be interpreted with caution. This delta part is known to be particularly important for rTSA function and, despite caution, may be injured via the deltopectoral approach [59, 68–71]. In the present study, TM atrophy was associated with inferior ROM. This is in line with previous findings that after rTSA, the infraspinatus and TM remain key external rotators [5, 72–74].

Synovitis is a known key pain mediator in osteoarthritis [75]. Various synovial patterns have been described post-arthroplasty, including infection or loosening, but also asymptomatic cases [14–16, 76]. In contrast to earlier studies, no MRI features of infection (lamellar synovitis, sinus tract, lymphadenopathy, extra-articular fluid) were observed in this cohort [77, 78]. Still, synovitis was common and linked to more pain and lower strength, and careful differentiation from artifacts or ossifications is critical.

Limitations of this study include the small cohort size, which limits generalizability. Second, only one postoperative time point was assessed. However, a 1-year interval minimizes confounding by early postoperative changes or late wear. Third, only in a subset of 13/52 individuals was preoperative MRI available. In the remaining cases, the indication for rTSA was made without the need for MRI. Fourth, only 10 CSSEMAC reconstruction iterations were feasible, due to long reconstruction times of the research software and limited computational power [14, 15]. Last, only CSSEMAC STIR sequences were diagnostically reasonable in the current setting.

In conclusion, this study offers normative MARS MRI data for patients 1 year after rTSA implantation. Understanding typical postoperative patterns, including muscle changes of the midacromial deltoid muscle, subacromial edema/fluid, small effusion, and minor BME, prevents overinterpretation and helps to distinguish pathological from physiological imaging findings.

## Supplementary Information

Below is the link to the electronic supplementary material.

ESM 1 Supplemental Material S1. (**a**) Schematic illustration of zones 1 to 7 according to the classification of Gruen adapted to the shoulder. (**b**) Schematic illustration of zones 1 to 6 according to the classification of Molé adapted for reverse total shoulder arthroplasties (Molé zones were originally described for anatomical total shoulder arthroplasties). Zone 1: fixation area of the superior part of the glenoid component base plate; Zone 2: fixation area of the superior part of the keel; Zone 3: fixation area of the tip of the keel; Zone 4: fixation area of the inferior part of the keel; Zone 5: fixation area of the inferior part of the glenoid component base plate; Zone 6: fixation area of the central part of the glenoid component base plate (PNG 114 KB)

High Resolution Image (TIFF 12.5 MB)

ESM 2 Supplemental Material S2. Flowchart of patient selection (PNG 84.9 KB)

High Resolution Image (TIFF 196 KB)

ESM3 Supplemental Material S3. Tables S3a and S3b (PDF 109 KB)

ESM 4 Supplemental Material S4. Overview of frequent MRI findings 1 year post rTSA implantation and overview of findings demonstrating significant associations with clinical Constant–Murley score (CMS) subscores pain, activities of daily living (ADL), range of motion (ROM), and strength in the multivariable regression models (PNG 859KB)

High Resolution Image (TIF 671 KB)

## Data Availability

Not applicable.

## Abbreviations

ATD: Acromion-tubercle distance
ADL: Activities of daily living
BMI: Body mass index
BME: Bone marrow edema
CS: Compressed sensing
CMS: Constant-Murley score
IMw: Intermediate weighted
ISP: Infraspinatus muscle
KL: Kellgren-Lawrence
LHO: Lateral humeral offset
MARS: Metal artifact reduction sequences
MRI: Magnetic resonance imaging
ROM: Range of motion
rTSA: Reverse total shoulder arthroplasty
SOAS: Shoulder osteoarthritis severity
STIR: Short τ inversion recovery
SEMAC: Slice encoding for metal artifact reducing techniques
SD: Standard deviation
SSC: Subscapularis muscle
T1w: T1 weighted
T2w: T2 weighted
TM: Teres minor muscle
THA: Total hip arthroplasty
VAT: View-angle tilting
