# Supplementary figures and images for: Metal-artifact reduced MR imaging for reverse shoulder arthroplasty: findings 1 year after surgery
Source: Skeletal Radiol. 2026 Jan 21;55(5):1087–100. doi: 10.1007/s00256-025-05121-y (PMC13018018; doi:10.1007/s00256-025-05121-y)

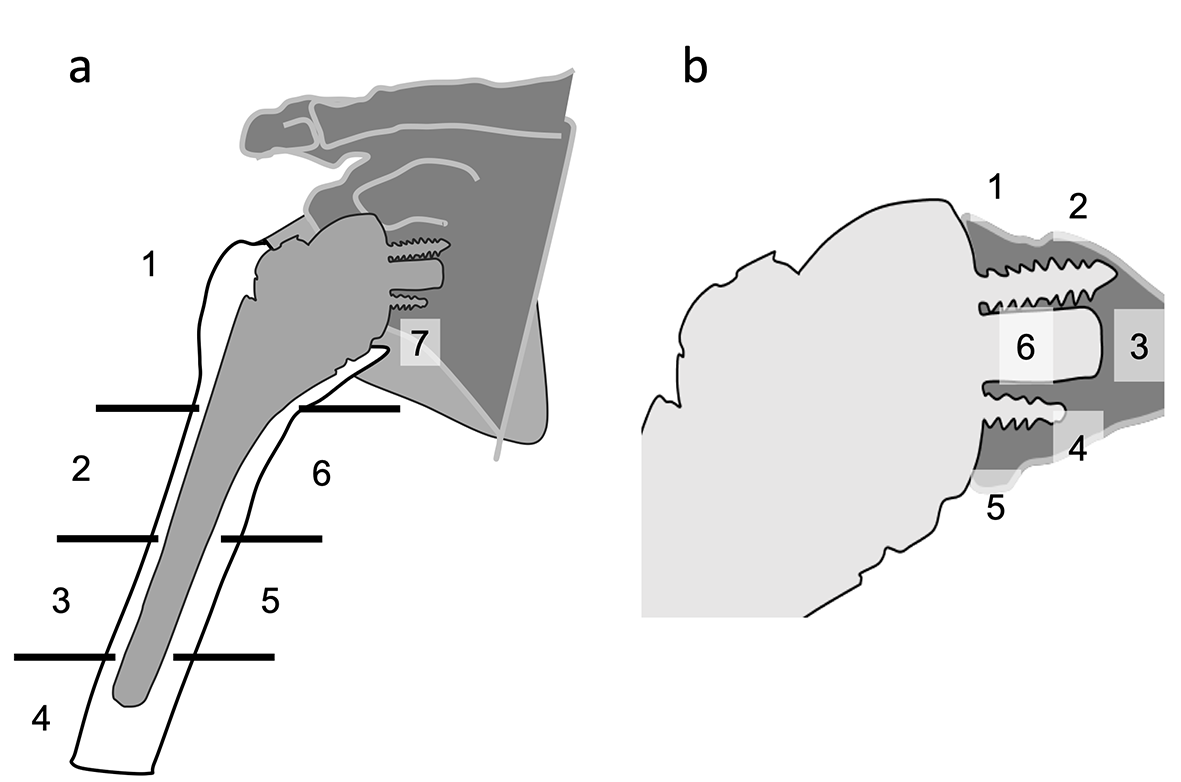

Supplement: Supplementary file 1 — Supplemental Material S1. (a) Schematic illustration of zones 1 to 7 according to the classification of Gruen adapted to the shoulder. (b) Schematic illustration of zones 1 to 6 according to the classification of Molé adapted for reverse total shoulder arthroplasties (Molé zones were originally described for anatomical total shoulder arthroplasties). Zone 1: fixation area of the superior part of the glenoid component base plate; Zone 2: fixation area of the superior part of the keel; Zone 3: fixation area of the tip of the keel; Zone 4: fixation area of the inferior part of the keel; Zone 5: fixation area of the inferior part of the glenoid component base plate; Zone 6: fixation area of the central part of the glenoid component base plate (PNG 114 KB) [file 256_2025_5121_Fig6_ESM.png]

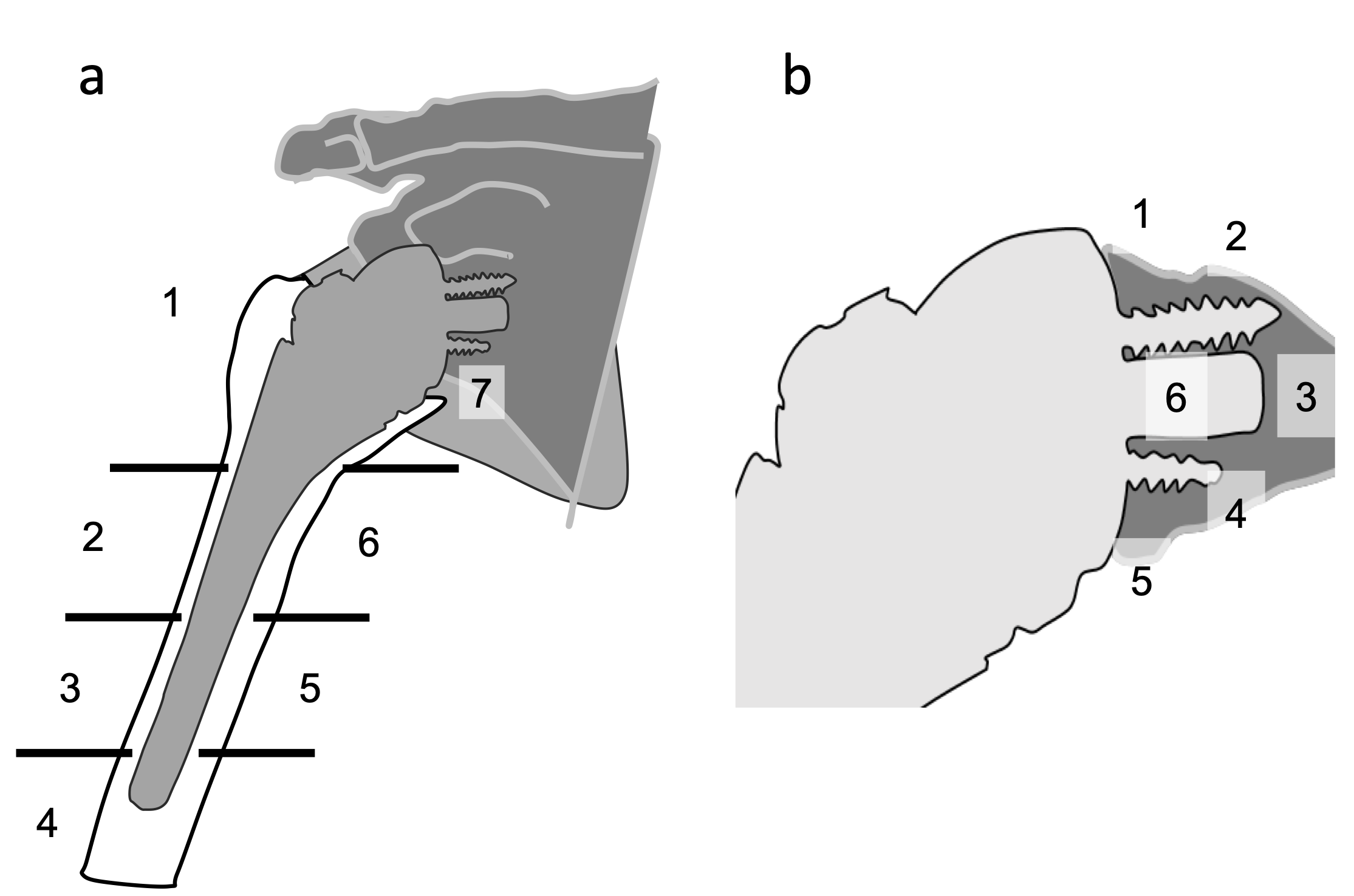

Supplement: Supplementary file 2 — High Resolution Image (TIFF 12.5 MB) [file 256_2025_5121_MOESM1_ESM.tif]

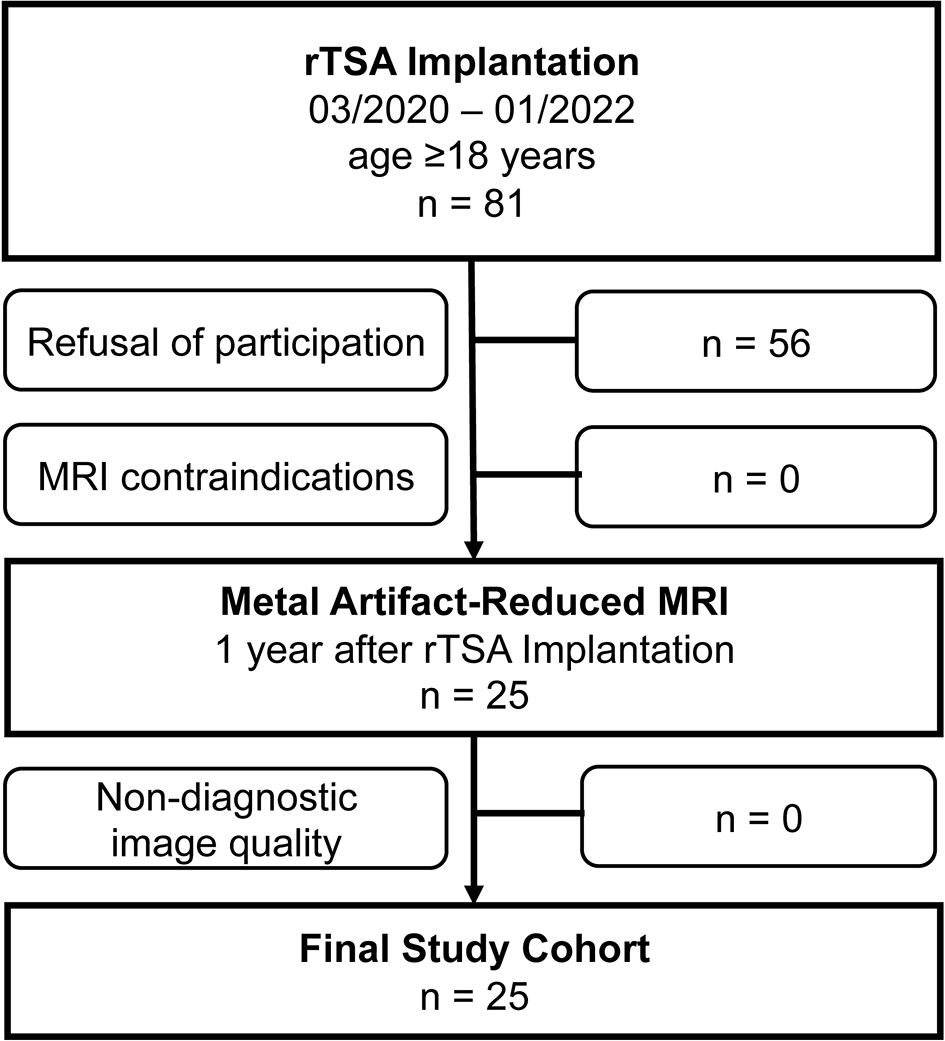

Supplement: Supplementary file 3 — Supplemental Material S2. Flowchart of patient selection (PNG 84.9 KB) [file 256_2025_5121_Fig7_ESM.png]

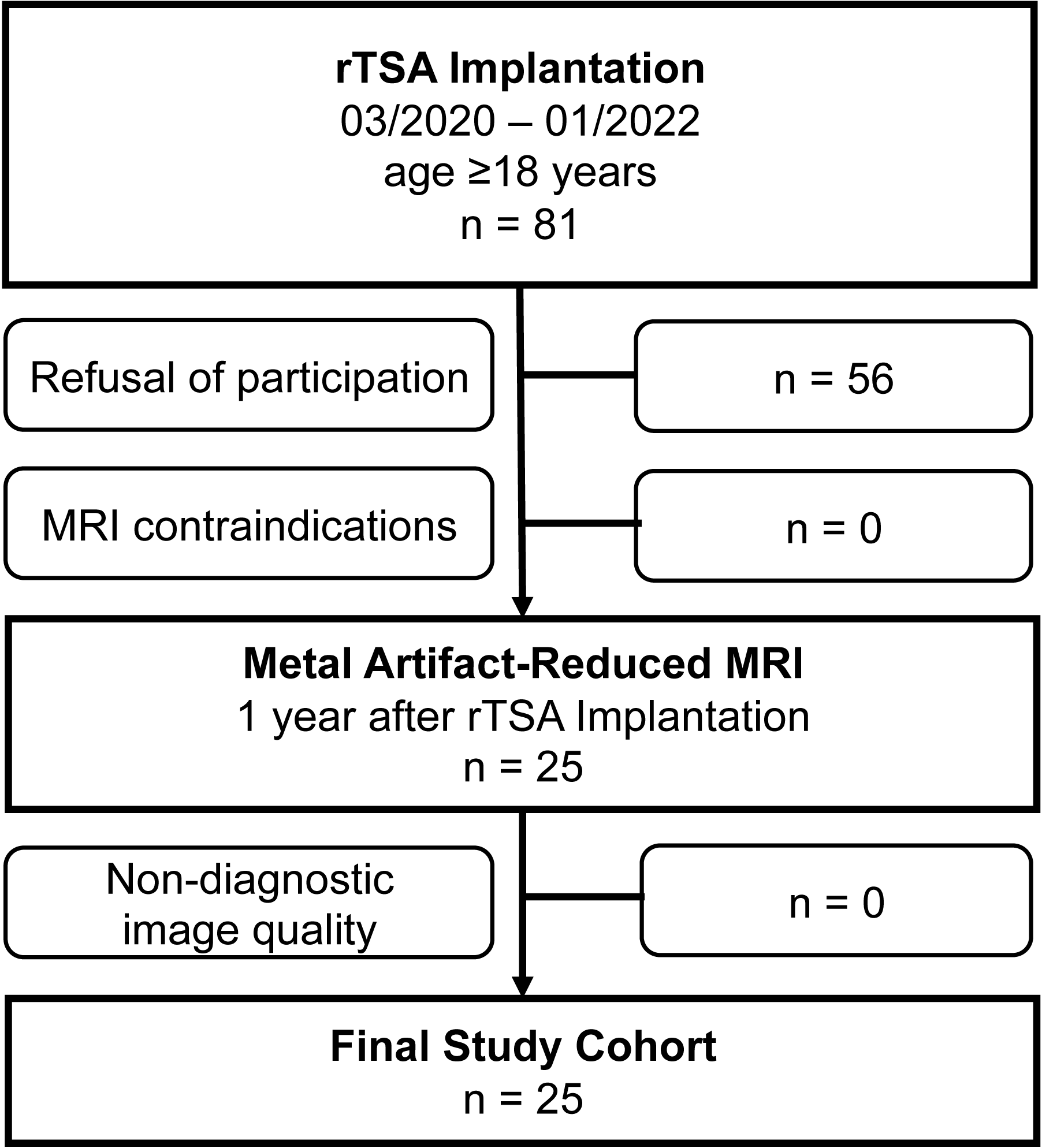

Supplement: Supplementary file 4 — High Resolution Image (TIFF 196 KB) [file 256_2025_5121_MOESM2_ESM.tif]

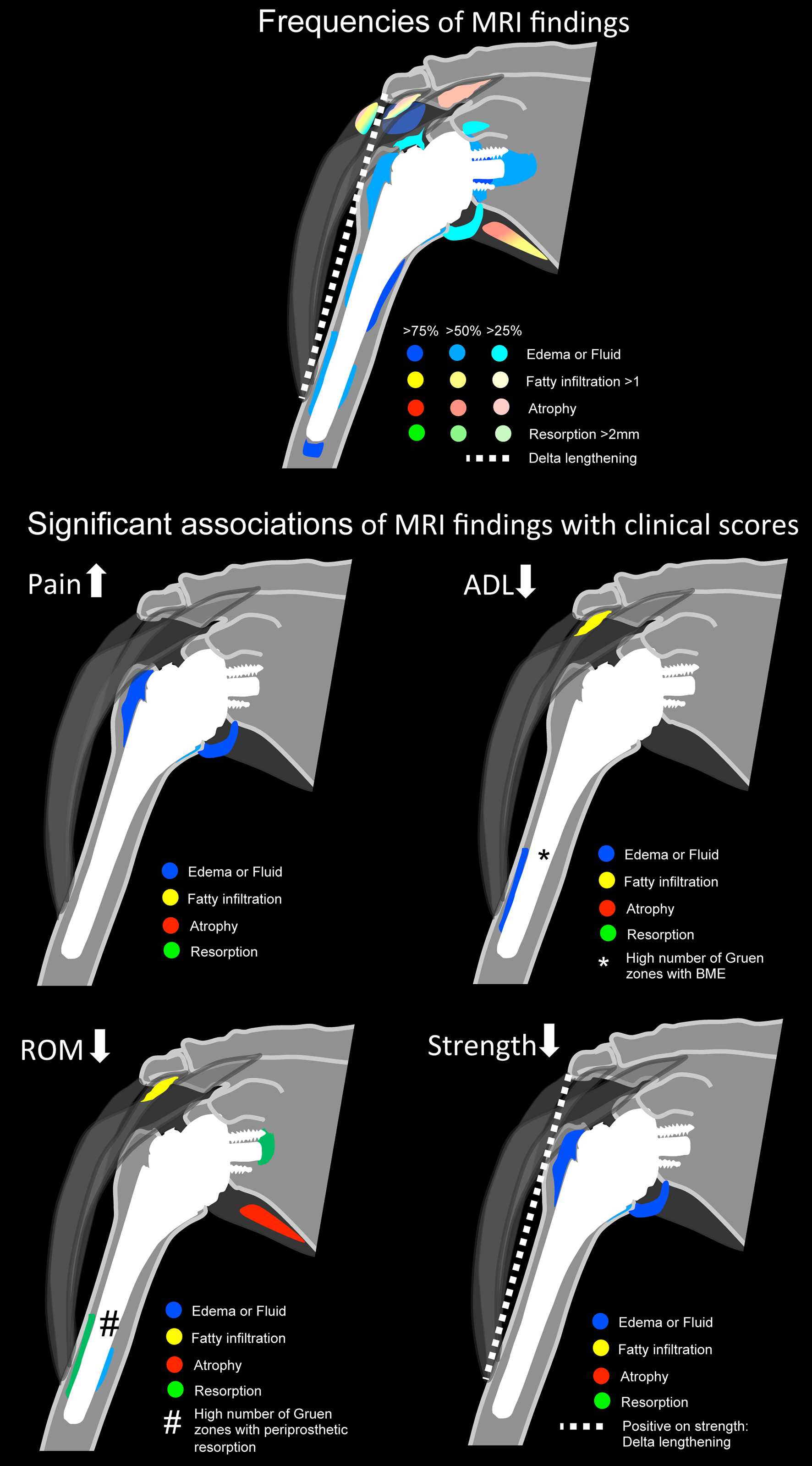

Supplement: Supplementary file 6 — Supplemental Material S4. Overview of frequent MRI findings 1 year post rTSA implantation and overview of findings demonstrating significant associations with clinical Constant–Murley score (CMS) subscores pain, activities of daily living (ADL), range of motion (ROM), and strength in the multivariable regression models (PNG 859KB) [file 256_2025_5121_Fig8_ESM.png]

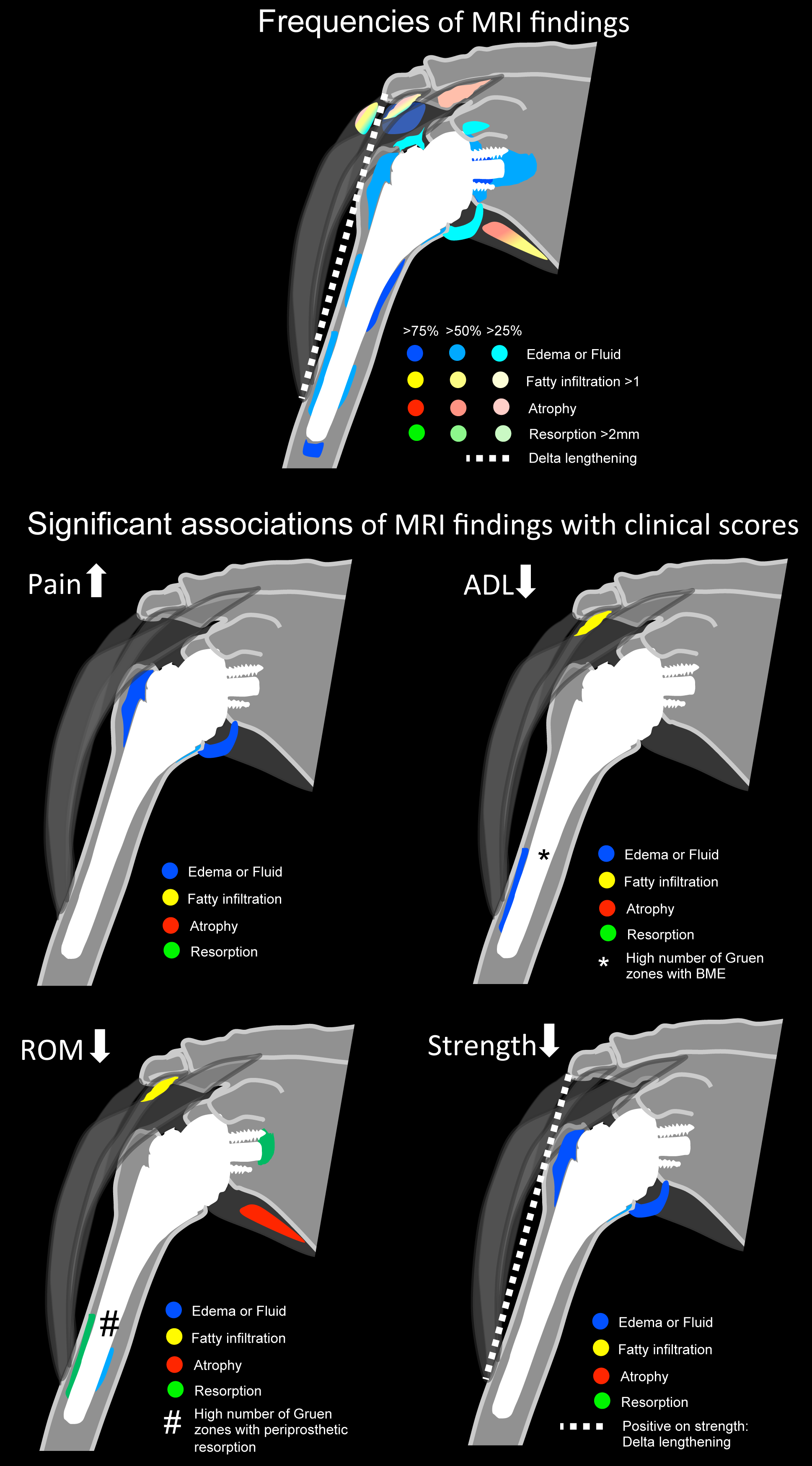

Supplement: Supplementary file 7 — High Resolution Image (TIF 671 KB) [file 256_2025_5121_MOESM4_ESM.tif]
