# Supplementary material for: Metal-artifact reduced MR imaging for reverse shoulder arthroplasty: findings 1 year after surgery
Source: Skeletal Radiol. 2026 Jan 21;55(5):1087–100. doi: 10.1007/s00256-025-05121-y (PMC13018018; doi:10.1007/s00256-025-05121-y)
Supplement: Supplementary file 5 — Supplemental Material S3. Tables S3a and S3b (PDF 109 KB) [file 256_2025_5121_MOESM3_ESM.pdf]

Supplemental Material S3

a. Table S3a. Frequency table for the evaluation of MARS MRI quality and artifacts on a 5-point Likert scale (1 worst, 5 best). MARS, metal artifact reducing sequences; CSSEMAC, compressed sensing slice encoding for metal artifact correction; STIR, short tau inversion recovery, VAT, view angle tilting. *P*-values are based on Wilcoxon Signed-Rank tests.

Parameter	Frequency (Percent) 1	2	3	4	5	*p*-value CSSEMAC versus VAT
Overall metal artifact reduction	0 (0%)	1 (4%)	12 (48%)	11 (44%)	1 (4%)	
Motion artifacts	0 (0%)	2 (8%)	3 (12%)	3 (12%)	17 (68%)	
Overall image quality	0 (0%)	1 (4%)	10 (40%)	14 (56%)	0 (0%)	
Ripple artifacts CSSEMAC STIR	0 (0%)	1 (4%)	13 (52%)	11 (44%)	0 (0%)	
Artifact reduction CSSEMAC STIR	0 (0%)	0 (0%)	5 (20%)	18 (72%)	2 (8%)	*p*<0.001
Artifact reduction VAT STIR	1 (4%)	11 (44%)	12 (48%)	1 (4%)	0 (0%)	
Soft tissue image quality CSSEMAC STIR	0 (0%)	0 (0%)	13 (52%)	12 (48%)	0 (0%)	*p*=0.346
Soft tissue image quality VAT STIR	1 (4%)	1 (4%)	5 (20%)	18 (72%)	0 (0%)	
Overall image quality CSSEMAC STIR	0 (0%)	0 (0%)	8 (32%)	15 (60%)	2 (8%)	*p*=0.001
Overall image quality VAT STIR	1 (4%)	4 (16%)	15 (60%)	4 (16%)	1 (4%)	

b. Table S3b. Intra- and interreader agreement. Intra- and interreader agreement was determined for the postoperative MRI findings. ICC, Interclass Correlation Coefficients.

Parameter	ICC scores (lower, upper 95% confidence interval) Interreader reliability	Intrareader reliability R1	Intrareader reliability R2
**Deltoid muscle fatty infiltration**	0.75 (0.53, 0.85)	0.79 (0.54, 0.89)	0.77 (0.63, 0.84)
**Deltoid muscle atrophy**	0.81 (0.70, 0.88)	0.85 (0.77, 0.90)	0.81 (0.70, 0.88)
**Deltoid muscle edema**	0.74 /0.59, 0.84)	0.81 (0.70, 0.88)	0.74 (0.59, 0.84)
**Bone marrow edema**	0.91 (0.89, 0.93)	0.95 (0.94, 0.96)	0.90 (0.89, 0.92)
**Periprosthetic resorption**	0.86 (0.83, 0.89)	0.86 (0.82, 0.89)	0.87 (0.84, 0.90)
**Periosteal edema**	0.80 (0.76, 0.84)	0.81 (0.77, 0.84)	0.83 (0.79, 0.86)
**Periosteal reaction**	Agreement close to 100%, ICC cannot be calculated (0.00)		
**Synovitis & Lamellar synovitis**	0.87 (0.75, 0.93)	0.88 (0.78, 0.93)	0.89 (0.79, 0.94)
**Subacromial changes (edema/ fluid)**	0.90 (0.83, 0.94)	0.95 (0.92, 0.97)	0.89 (0.81, 0.94)
